# Advanced Li–S Battery Configuration Featuring Sulfur‐Coated Separator and Interwoven rGO/CNT Fabric Current Collector

**DOI:** 10.1002/smll.202405365

**Published:** 2024-10-29

**Authors:** Kuan‐Cheng Chiu, Asif Latief Bhat, Ching‐Kuan Yang, Sheng‐Heng Chung, Niall Tumilty, Yu‐Sheng Su

**Affiliations:** ^1^ International College of Semiconductor Technology National Yang Ming Chiao Tung University 1001 Daxue Road Hsinchu City 300093 Taiwan; ^2^ Industry Academia Innovation School National Yang Ming Chiao Tung University 1001 Daxue Road Hsinchu City 300093 Taiwan; ^3^ Department of Materials Science and Engineering National Cheng Kung University No.1 University Road Tainan City 70101 Taiwan

**Keywords:** active sulfur redistribution, carbon nanotubes, energy storage, polysulfide adsorption, reduced graphene oxide, separator coating

## Abstract

The development of lithium–sulfur batteries (LSBs) marks a crucial milestone in advancing energy storage solutions essential for sustainable energy transitions. With high theoretical specific capacity, cost‐effectiveness, and reduced ecological footprint, LSBs promise to enhance electric vehicle ranges, extend portable electronics' operational times, and stabilize grids integrated with renewable energy. However, challenges like complex processing, electrode instability, and poor cycling stability hinder their commercialization. This study introduces a novel battery design that addresses these issues by coating sulfur directly onto the separator instead of the current collector, demonstrating that active sulfur can be effectively utilized without being incorporated into the electrode structure. Using an interwoven substrate made from carbon nanotube (CNT) fabric adorned with reduced graphene oxide (rGO), this setup enhances manufacturing scalability, supports optimal sulfur utilization, and improves battery performance. The rGO decoration provides multiple highly conductive polysulfide trapping sites, enhancing active material reutilization, while the flexibility and mechanical strength of CNT fabric contribute to electrode integrity. This combination boosts electrical conductivity and polysulfide‐capturing capability, effectively managing migrating sulfur species during charge–discharge cycles and mitigating sulfur loss and polysulfide shuttling. The results demonstrate superior cycling stability and efficiency, highlighting the potential of this approach in advancing LSB technology.

## Graphical abstract

This study presents an innovative lithium–sulfur battery (LSB) design where sulfur is directly coated onto the separator instead of the electrode, eliminating the cumbersome synthesis process. An interwoven carbon nanotube (CNT) fabric with reduced graphene oxide (rGO) decoration enhances conductivity and polysulfide trapping. This novel configuration significantly boosts cycling stability and scalability, advancing LSB technology for energy storage applications.

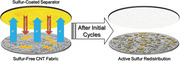

## 1 Introduction

The quest for advanced energy storage solutions has never been more pressing, driven by the imperative to transition toward sustainable energy sources and mitigate environmental impact. Lithium–sulfur batteries (LSBs) have emerged as promising candidates due to their high theoretical specific capacity, low‐cost potential, and reduced environmental footprint compared to conventional lithium‐ion technologies.^[^ ^1^, ^2^ ^]^ The ability of LSBs to potentially deliver significantly higher energy densities promises extended range and improved performance for electric vehicles, prolonged operation times for portable electronics, and enhanced grid stability for renewable energy integration. However, to realize these advantages at a commercial scale, overcoming challenges such as active material loss, electrode instability, and poor cycle life remains paramount. The landscape of LSBs has seen significant innovation in recent years, marked by various configurations such as interlayers,^[^ ^3^, ^4^, ^5^ ^]^ carbon‐coated separators,^[^ ^5^, ^6^, ^7^, ^8^ ^]^ and polysulfide catholytes.^[^ ^9^, ^10^, ^11^ ^]^ Traditionally, the emphasis has been on integrating sulfur directly into the cathode structure through methods like sulfur infiltration or the use of polysulfide solutions. These approaches aim to enhance the battery energy density by leveraging sulfur's high theoretical capacity. However, some traditional methods pose challenges including poor scalability, cycle life degradation, and safety concerns associated with polysulfide handling. Remarkably, despite extensive research into enhancing LSB performance, little attention has been given to sulfur‐free electrode designs until now.

**Scheme 1:**
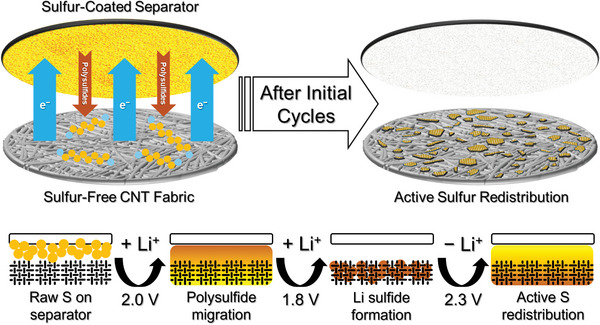
The configuration and working mechanism of the sulfur‐free CNT membrane with a sulfur‐coated separator design for Li‐S batteries.

This study employs an innovative battery design where sulfur is no longer incorporated into the cathode. Instead, sulfur is directly coated onto the separator, as illustrated in **Scheme** **1**. The cathode structure is made exclusively of sulfur‐free carbon nanotube (CNT) interwoven fabric, which is specifically decorated with graphene oxide (GO) or reduced graphene oxide (rGO) to improve interfacial performance. During the electrochemical reaction, the insulating sulfur coated on the separator interacts with the CNT‐supported current collector to facilitate electron transfer, thereby reacting with lithium ions, which then dissolve in the electrolyte and migrate into the cathode structure. Active sulfur species are transported to reactive sites, undergoing dynamic redistribution throughout cycling. This approach contrasts with traditional LSB designs, which typically require pre‐mixing sulfur into the cathode in various forms. Conventional methods include the high‐temperature process of sulfur infiltration into the cathode,^[^ ^12^ ^]^ infusing the cathode with highly reactive polysulfide solutions,^[^ ^9^, ^13^ ^]^ or synthesizing pure sulfur as the active material using sodium thiosulfate and corrosive hydrochloric acid.^[^ ^14^, ^15^ ^]^ These conventional techniques necessitate additional materials and increase manufacturing costs and safety concerns. In comparison, this configuration is not only cost‐effective but also enhances cycle performance. Furthermore, the CNT fabric is fabricated in a free‐standing manner without the need for binders.^[^ ^3^, ^16^, ^17^ ^]^ This improves the energy density and eliminates the negative impact of commonly used insulating polymer binders on the overall conductivity.

## 2 Results and Discussion

**Figure 1:**
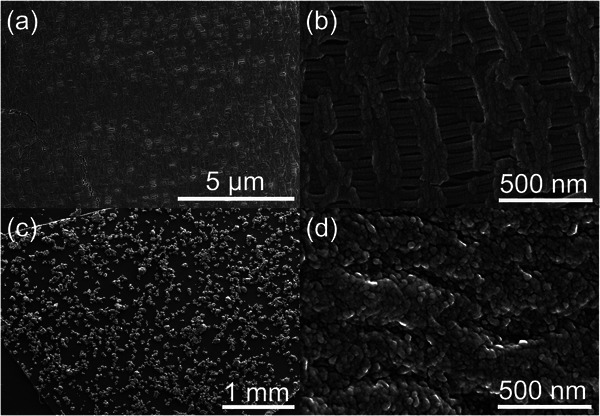
Scanning electron microscopy (SEM) images of the separator a,b) before coating and c,d) after sulfur coating.

The surface morphology of the separator before and after coating is shown in **Figure** **1**a,b, which represent a commercially available Celgard separator, which is a three‐layer membrane composed of polypropylene (PP) and polyethylene (PE). The surface features fine pores that allow ion exchange during electrochemical reactions. For comparison, the separator image after sulfur coating is magnified in Figure 1c,d. Since sulfur does not dissolve in NMP but forms a suspension, numerous sulfur particles are attached to the separator surface rather than forming a continuous sulfur layer. It is worth noting that the coated sulfur on the separator is the only source of active material in the developed LSBs.

**Figure 2:**
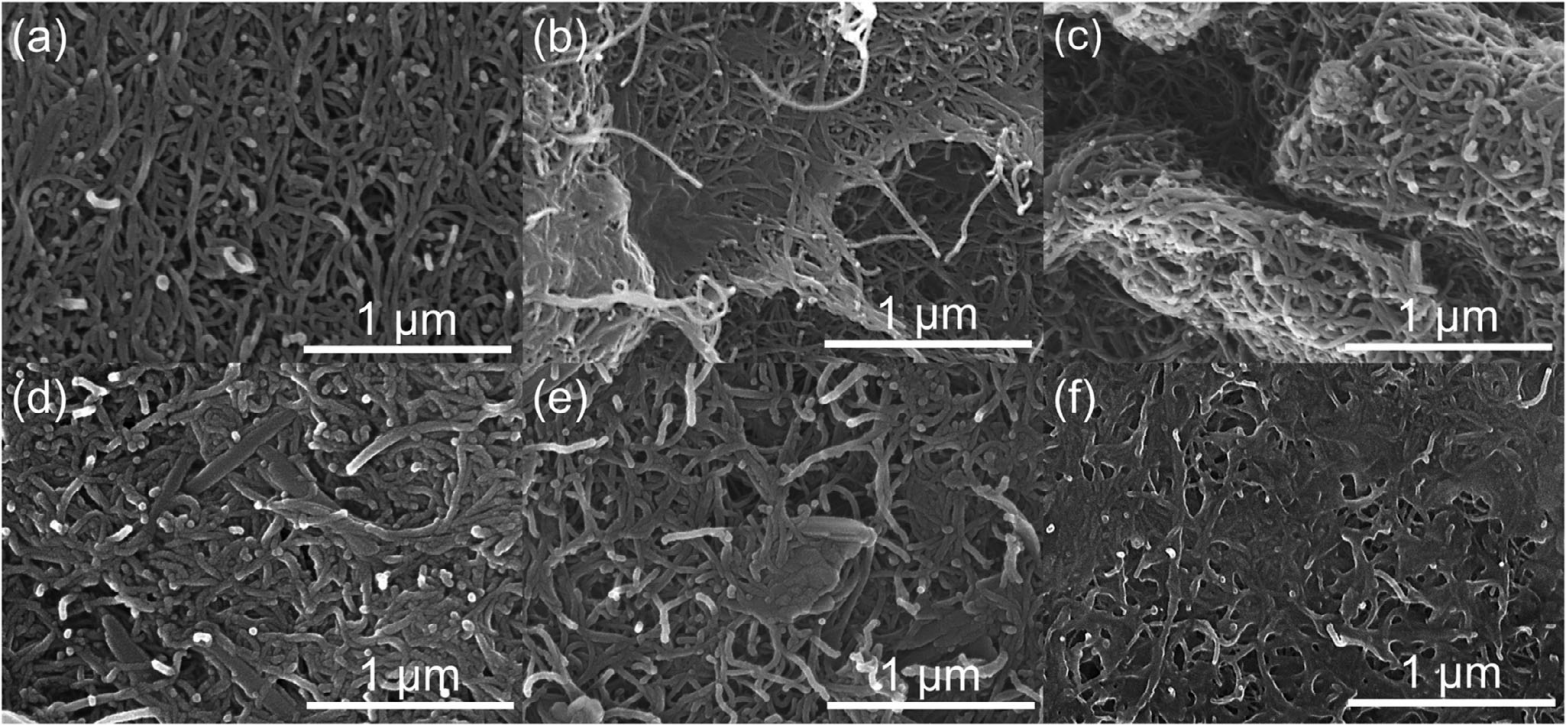
SEM images of different CNT‐supported current collectors with sulfur‐coated separators (SCS) before cycling: a) CNT/SCS, b) CNT+GO/SCS, and c) CNT+rGO/SCS, and after cycling: d) CNT/SCS, e) CNT+GO/SCS, and f) CNT+rGO/SCS.

**Figures** **2** and S1 (Supporting Information) show the microstructural morphology of each CNT‐supported current collector before and after cycling. The pristine CNT membrane exhibits a planar structure with entangled nanotubes with outstanding flexibility and mechanical strength after film formation (Figure 2a). This structure, created through a simple vacuum filtration method with ultrasonic agitation, has numerous pores that not only buffer the volume changes of sulfur during cycling but also provide ample space for the accommodation of soluble polysulfides.^[^ ^3^, ^17^ ^]^ Figure 2b,c reveal that when GO and rGO are added to the CNTs, the CNT web is coated with 2D graphene sheets, showing a film‐like structure. This structural variation provides additional adsorption sites for sulfur, while the added GO and rGO sheets have strong inherent adsorption capability for dissolved polysulfides.^[^ ^18^ ^]^ After battery cycling, as shown in Figure 2d–f, the diameters of CNTs significantly increase. This is due to the adsorption of soluble active sulfur species migrating from the separator onto each nanotube, resulting in a more compact electrode structure. This demonstrates the effectiveness of the interwoven CNT+rGO fabric in adsorbing dissolved polysulfides. Additionally, the detection of active materials in the CNT‐supported current collectors indicates the excellent conductivity of these 3D current collectors, facilitating the electrochemical reaction of the sulfur material coated on the separator, which dissolves into the electrolyte and migrates to the cathode region after initial cycles.

To gain a deeper understanding of the changes introduced by incorporating GO and rGO into CNT‐supported current collectors, Raman analysis was performed on these materials (Figure S2, Supporting Information). The characteristic peaks of carbon‐based materials appear ≈1350 cm⁻^1^ for the D‐band, which represents defects and disorder in the carbon lattice, and ≈1580 cm⁻^1^ for the G‐band, which indicates the degree of graphitization.^[^ ^19^, ^20^ ^]^ The intensity ratio of the D‐band to the G‐band (I_D_/I_G_) is commonly used as an indicator of graphene defect density.^[^ ^19^, ^20^ ^]^ All the D‐band and G‐band peak locations are nearly overlapping, with differences mainly in the I_D_/I_G_ value. Although thermally reducing GO to rGO removes surface functional groups and increases defects, high‐temperature treatment of CNTs shows a more influential effect. Previous experimental results indicate that as the temperature increases, defects in CNTs decrease, leading to a more ordered graphitic structure, which lowers the I_D_/I_G_ ratio in Raman spectra.^[^ ^16^, ^21^ ^]^ This is because high temperatures facilitate the rearrangement of carbon atoms within the sp^2^ plane, enhancing crystallinity. In the current collector design, the dominant presence of CNTs in the composite materials means that the properties of CNTs have a greater influence on the overall material characteristics than the rGO. Therefore, despite the introduction of more defects through thermal reduction to generate rGO, the high‐temperature treatment characteristics of CNTs result in the overall Raman spectrum showing fewer defects. Conversely, the CNT+GO without heat treatment exhibits a significantly higher I_D_/I_G_ ratio.

**Figure 3:**
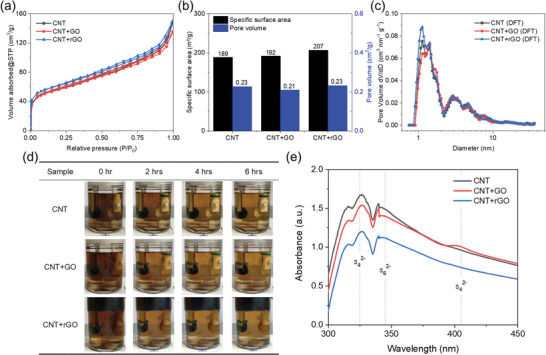
a) Nitrogen adsorption/desorption isotherms, b) specific surface area and pore volume, and c) pore size distribution curves of CNT‐supported current collectors. d) Color change of polysulfide solution with different CNT‐supported current collectors during electrochemical cell reactions at different times, and e) UV–vis spectra after 24 h of reaction.

The nitrogen adsorption‐desorption isotherms for pristine CNT and surface modified CNT materials are shown in **Figure** **3**a. All three materials exhibit Type II adsorption‐desorption curves, also known as S‐shaped isotherms.^[^ ^22^, ^23^ ^]^ This type of curve typically occurs in non‐porous or macroporous solids and represents a reversible multilayer adsorption process. Among the curves, the CNT+rGO composite shows the highest curve value, indicating that the reduction of graphene oxide generates more interfacial defects, leading to the highest surface area among all the samples. Further analysis using the Brunauer–Emmett–Teller (BET) model reveals the specific surface areas of CNT, CNT+GO, and CNT+rGO to be 189, 192, and 207 m^2 ^g^−1^, respectively (Figure 3b). These results demonstrate that the inclusion of graphene nanosheets indeed increases the specific surface area, with the CNT+rGO showing the greatest enhancement. The curves in Figure 3c obtained using the density functional theory (DFT) model reveal that the pore size distributions of CNT, CNT+GO, and CNT+rGO are mostly below 10 nm, with pore volumes of 0.23, 0.21, and 0.23 cm^3^ g^−1^, respectively (Figure 3b). Micropores contribute significantly to the overall pore volume, with CNT+rGO demonstrating the highest value among the three materials, while CNT+GO shows a slightly smaller pore volume. These results indicate that the composite structure of CNT+rGO, with its high specific surface area, provides more channels for the soluble polysulfides to flow in more thoroughly. This also promotes Li‐ion transport, thereby enhancing the stability and battery cycle life.

A transparent electrochemical cell and a polysulfide solution were employed to observe the polysulfide adsorption ability during discharge, with lithium metal at one end and the CNT‐supported current collectors at the other. Changes in polysulfide concentration are determined by color change, as shown in Figure 3d. Visually, the pristine CNT current collector displays the deepest yellow‐brown color after 6 h. In contrast, the CNT+GO and CNT+rGO current collectors show lighter colors post‐reaction. Since the color was not easily discernible to the human eye, these reacted electrolytes were further examined using UV–VIS spectroscopy. The results, shown in Figure 3e, indicate that the pristine CNT fabric exhibited the highest signal intensity, corresponding to its darker color. Across all wavelength ranges, the absorbance of CNT+rGO is lower than that of CNT+GO interwoven fabric, indicating better reutilization and conversion of polysulfides. Additionally, a peak ≈400 nm in the CNT+GO spectrum corresponds to polysulfide S_4_^2−^,^[^ ^24^ ^]^ indicating that the CNT+GO current collector is less effective at adsorbing polysulfides containing S_4_^2−^ compared to the other two substrates. As a result, CNT+rGO demonstrates superior polysulfide adsorption capacity compared to the other two materials.

**Figure 4:**
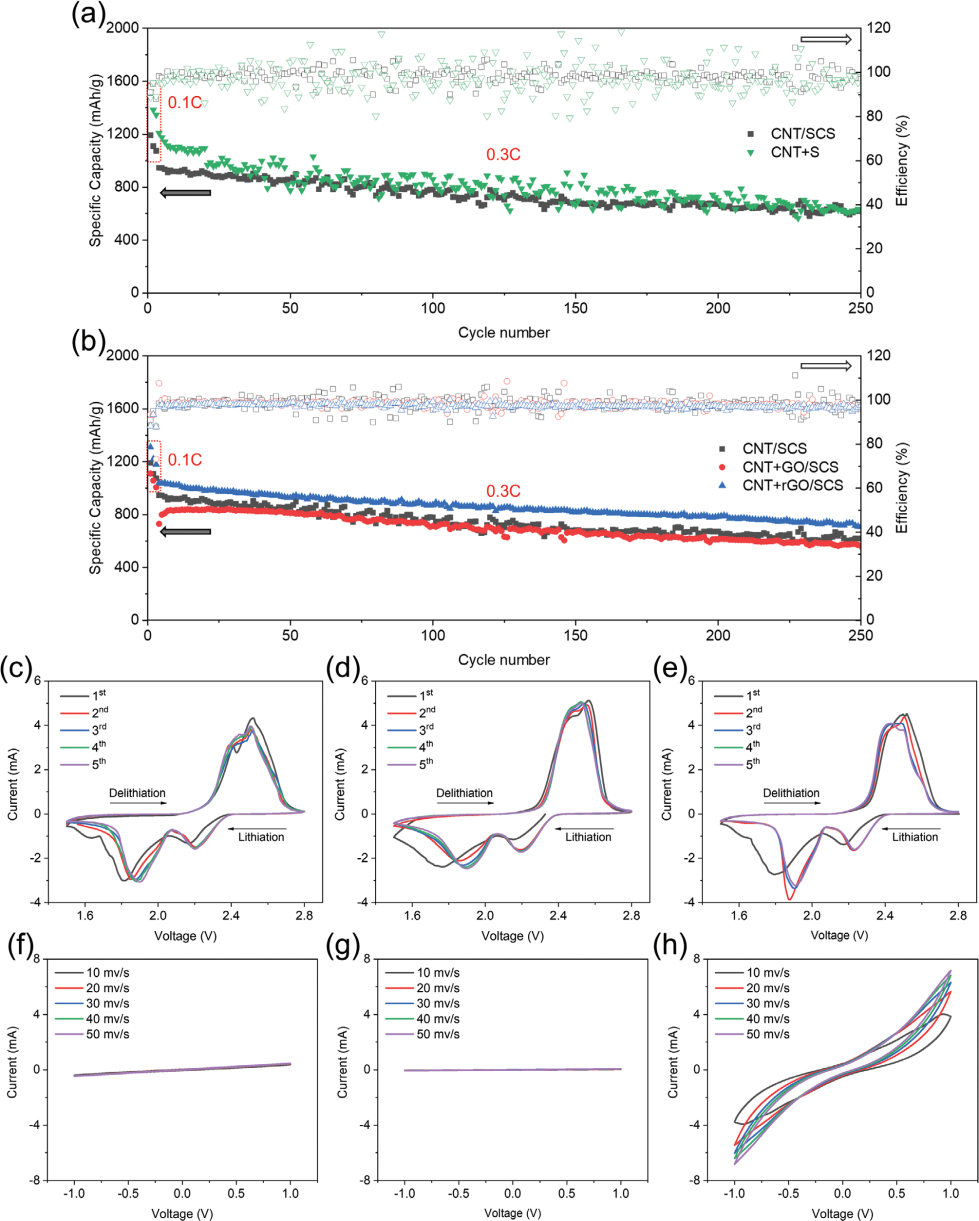
a) Cycle life comparison of a sulfur‐free CNT current collector with the sulfur‐coated separator (CNT/SCS) and a sulfur‐loaded CNT electrode (CNT+S) and b) cycle life with the sulfur‐coated separator and different CNT‐supported current collectors. CV curves of Li‐S batteries with c) CNT/SCS, d) CNT+GO/SCS, and e) CNT+rGO/SCS configurations at a scan rate of 0.1 mV s^−1^. CV curves of symmetric cells with f) CNT, g) CNT+GO, and h) CNT+rGO current collectors.

To compare the impact of sulfur placement in the LSB configuration, the differences between a conventional cathode structure containing sulfur and the developed structure with sulfur coated on the separator were investigated. For the conventional cathode, sulfur powder was mixed with CNT powder, dispersed through ultrasonication, and then vacuum filtered and dried to form a freestanding CNT+S electrode with the same active material loading of 2.0 mg cm^−2^. As evident in **Figure** **4**a, the advanced cell configuration with a sulfur‐coated separator design does not compromise cycle stability compared to the conventional method. Although the conventional CNT+S cathode achieves an initial capacity of over 1521 mAh g^−1^, its overall cycle performance and Coulombic efficiency are unstable, fluctuating between 1000 and 700 mAh g^−1^, with a capacity retention rate of 49% after 250 cycles at a 0.3C rate. In contrast, the sulfur‐free electrode with a sulfur‐coated separator design (CNT/SCS) shows a smooth and stable cycling curve, with a capacity retention rate of 62% after 250 cycles at the same C‐rate. This demonstrates that the novel battery configuration, with sulfur coated on the separator, achieves better performance in terms of lifespan and capacity/efficiency stability in LSBs compared to the traditional design.

Next, cycling tests were conducted using sulfur‐coated separators with three different CNT‐supported current collectors under identical conditions. Figure 4b shows that the capacity of the LSB using CNT+GO fabric decreases slightly compared to the pristine CNT fabric. This decrease could be attributed to the numerous functional groups and defects in GO, which lead to an insulating oxide layer and surface defects that reduce electrical conductivity, resulting in low active material utilization. Consequently, this leads to lower cycling capacity and Coulombic efficiency, likely due to irreversible side reactions between the functional groups and active lithium ions. On the other hand, the substrate formed by combining CNTs with rGO exhibits the best cycle life. This structure not only achieves a high capacity of 1310 mAh g^−1^ in the initial cycle but also demonstrates the smoothest and most stable overall cycling curve. After high‐temperature reduction, the oxide layer in GO is removed, significantly reducing rGO's resistivity. Moreover, the defects in rGO formed during the reduction process increase polysulfide capture, allowing the CNT+rGO fabric to provide higher and more stable capacity and Coulombic efficiency during cycling and exhibit a longer lifespan, achieving 70% capacity retention after 250 cycles. While adding an additional layer of CNT+rGO current collector initially increases the specific capacity based on sulfur mass, the cycle stability is compromised (Figure S3a, Supporting Information), and the overall energy density of the Li‐S cell is reduced due to the higher C/S ratio. Higher sulfur loadings on the coated separators were also evaluated (Figure S3b, Supporting Information), showing that higher active material loadings result in lower utilization efficiency due to reduced conductivity from the lower C/S ratio. Specifically, it took 8 cycles for the sample with 2.6 mg cm^−2^ sulfur loading and 52 cycles for the sample with 3.2 mg cm^−2^ sulfur loading to reach a stable capacity. Therefore, the subsequent data presented in this study are based on the single‐layer fabric design with a sulfur loading of 2.0 mg cm^−2^ to provide the optimal balance for battery performance. To understand the redox reactions occurring during cycling, CV tests were used to identify and analyze the reaction potential peaks. During the first discharge cycle, the LSBs exhibit two lithiation peaks at ≈2.3 and 1.8 V, corresponding to the conversion of sulfur (S_8_) to long‐chain polysulfides (Li_2_S_n_, 4 ≤ n ≤ 8) and short‐chain polysulfides (Li_2_S_2_/Li_2_S), respectively (Figure 4c–e).^[^ ^25^, ^26^, ^27^ ^]^ During the delithiation process, two oxidation peaks appear at ≈2.4 and 2.5 V, indicating the conversion of lithium sulfide to long‐chain polysulfides and then to sulfur.^[^ ^25^, ^26^ ^]^ In the first cycle, the redox peaks occur at slightly delayed positions due to the initial activation of the battery to dissolve the active sulfur coated on the separator. The second lithiation peaks ≈1.8 V in the CV profiles result from limited sulfur utilization, caused by restricted contact between the sulfur coating and the current collector, leading to higher overpotential. As cycling continues, active sulfur gradually migrates to the current collector, reducing the overpotential and shifting the second lithiation peak toward 2.0 V. Both the oxidation and reduction peaks shift to earlier positions in subsequent cycles, indicating smoother reaction kinetics after the activation of the LSB, promoted by the redistributed active material in the CNT framework. The overlapping of the peaks also demonstrates good electrochemical reaction reversibility. Among the three CNT‐supported current collectors, the CNT+rGO shows a higher reduction in peak intensity, mainly due to its larger specific surface area, higher porosity, and better conductivity.^[^ ^4^ ^]^ The defects on the rGO surface provide more sites for the adsorption and reutilization of polysulfides in the cathode region. These characteristics give the CNT+rGO fabric significant advantages in electrochemical performance.

To evaluate the electrochemical reactivity of CNT‐supported current collectors in LSBs, symmetric cells were assembled with a 0.05 M polysulfide catholyte as the active material. CV tests were conducted at scan rates ranging from 10 to 50 mV s^−1^ within a potential range of −1–1 V. Figure 4f–h shows that with increasing scan rate, the redox current of CNT+rGO fabric gradually increases and is dramatically higher than that of the other two substrates. This indicates that CNT+rGO fabric exhibits superior lithium polysulfide/sulfide redox kinetics.^[^ ^28^ ^]^ In contrast, the current response of CNT+GO was the lowest. This may be due to the presence of numerous functional groups in GO. While the defects on GO sheets help immobilize polysulfides, the insulating nature of oxygen‐containing functional groups reduces conductivity, affecting the overall electrochemical performance. GO cannot provide as excellent electron conduction pathways as rGO, leading to a poorer response in redox reactions.

**Figure 5:**
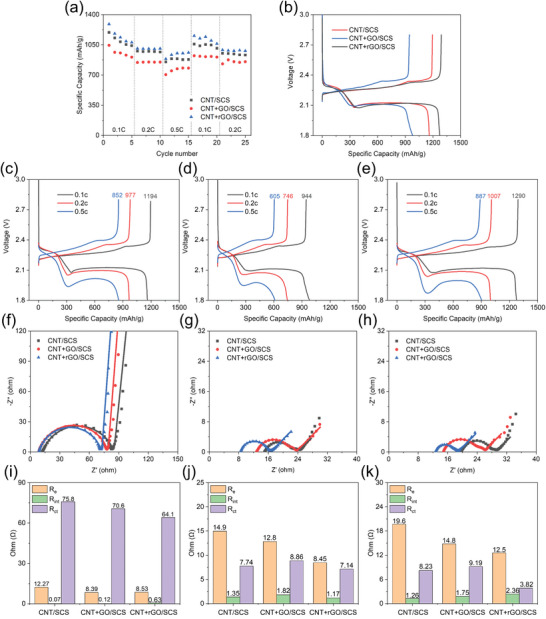
a) Rate performance of different CNT‐supported current collectors with sulfur‐coated separators, b) charge–discharge curves at 0.1C for different CNT‐supported current collectors with sulfur‐coated separators, and charge–discharge curves at various rates for c) CNT/SCS, d) CNT+GO/SCS, e) CNT+rGO/SCS configurations. EIS comparison of different CNT‐supported current collectors with sulfur‐coated separators f) before cycling, g) after 50 cycles, and h) after 100 cycles. Impedance analysis of different CNT‐supported current collectors with sulfur‐coated separators i) before cycling, j) after 50 cycles, and k) after 100 cycles.

In **Figure** **5**a, it is evident that the CNT+rGO interwoven fabric consistently exhibits superior performance across all charge–discharge rates. At a rate of 0.5C, the capacity remains ≈900 mAh g^−1^. In comparison, the CNT and CNT+GO current collectors demonstrate lower overall capacities than CNT+rGO, indicating a certain degree of irreversibility and inactivity. As shown in Figure 5b–e, the charge–discharge curves of CNT‐supported current collectors at different rates clearly illustrate that the overpotential of redox reactions increases with current density. Notably, the upper plateau of CNT+GO composite (Figure 5d) is significantly shorter than that of the other two current collectors. This suggests a lower proportion of sulfur converted to long‐chain polysulfides, likely due to the poorer conductivity of the GO coating, which hinders complete active material utilization. In the CNT+rGO design, CNTs provide excellent conductivity, while rGO enhances performance by contributing a high surface area, structural defects, and functionalized sites, all of which are essential for effectively trapping polysulfides.^[^ ^29^, ^30^, ^31^, ^32^ ^]^

Figure 5f–h shows the Nyquist plots of CNT‐supported current collectors before cycling, after 50 cycles, and after 100 cycles, respectively. The spectra were fitted using the simulated equivalent circuit shown in Figure S4 (Supporting Information).^[^ ^33^ ^]^ Here, R_e_, R_int_, and R_ct_ represent the electrolyte resistance, the interfacial resistance formed by the insoluble Li_2_S_2_/Li_2_S layer, and the charge transfer resistance at the electrode/electrolyte interface, respectively.^[^ ^33^ ^]^ CPE1 represents the interfacial capacitance, CPE2 represents the electrode/electrolyte capacitance, and W_0_ represents the Warburg impedance.^[^ ^33^ ^]^ The diameter of the semicircles before cycling is significantly larger than after cycling, where the R_ct_ for CNT+GO is higher than for CNT+rGO due to its poorer electrical conductivity, which increases the charge transfer resistance after cycling. Sulfur dissolves and enters the CNT‐supported interwoven fabric, redistributing the material to an optimal position, resulting in a more stable system and a significant decrease in R_ct_ after activation. Detailed resistance changes over cycling are shown in Figure 5i–k, where R_ct_ decreases sharply. Next, a notable difference from conventional LSBs systems is observed due to the unique battery configuration in this study, where the active material, pure sulfur, is stored on the separator surface. During initial cycling, the conversion to soluble polysulfides and their migration through the electrolyte to the CNT‐supported current collectors significantly alters the electrolyte composition. The differences in R_e_ values reflect the varying polysulfide adsorption capabilities of current collectors, with CNT+rGO exhibiting the smallest R_e_ after cycling, indicating the best polysulfide adsorption ability, thus suppressing electrolyte degradation. The introduction of rGO further increases surface defect sites with superior electrical conductivity, effectively adsorbing polysulfides, performing complete redox reactions, and preventing their diffusion back into the electrolyte.

Post‐cycling morphological and compositional analysis was conducted using SEM on the cycled separators/Li electrodes and X‐ray photoelectron spectroscopy (XPS) depth profiling on all CNT‐supported current collectors with sulfur‐coated separators. As shown in Figure S5a–c (Supporting Information), optical images of the cycled separators reveal that the separator with the CNT+rGO configuration displays a significantly thicker yellow film, indicating higher sulfur reactivity during cycling. The SEM images (Figure S5d–i, Supporting Information) further confirm that the CNT+rGO/SCS configuration promotes efficient sulfur utilization, leading to a dense precipitate formation at the current collector/separator interface. Notably, the absence of original sulfur particles on the separator (Figure S5d,g, Supporting Information) suggests their complete dissolution during the initial cycles. Figure S6 (Supporting Information) presents a comparison of the cycled Li electrodes, revealing that the electrode paired with the CNT/SCS configuration exhibits loosely packed grains and large cracks, which could contribute to higher resistance. In contrast, the cycled Li electrode with the CNT+rGO/SCS configuration appears much denser, with smaller grains and no noticeable cracks, indicating better structural integrity and potentially lower resistance.

In Figure S7a,d,g (Supporting Information) , the C 1s spectra show a main characteristic peak at 284.5 eV. It is observed that the surface signals of the three CNT‐supported fabrics are the strongest, with the signal intensity decreasing and stabilizing at deeper levels. This is because the surface contains the most carbon species, such as decomposed electrolyte by‐products from the cycling process. The C─N bond signals originate from the electrolyte salt (LiTFSI) precipitated after sample drying.^[^ ^34^ ^]^ In Figure S7b,e,h (Supporting Information) , the Li 1s spectra show that the peak signals increase with etching depth, with peaks corresponding to Li─S bonds, representing lithium polysulfide and lithium sulfide signals.^[^ ^35^, ^36^ ^]^ This indicates that during the cycling process, polysulfides gradually penetrate and are captured in the deeper structure of CNT substrates. This suggests that the material can effectively trap polysulfides, preventing their diffusion, thus reducing active polysulfide loss and the shuttle effect.

**Figure 6:**
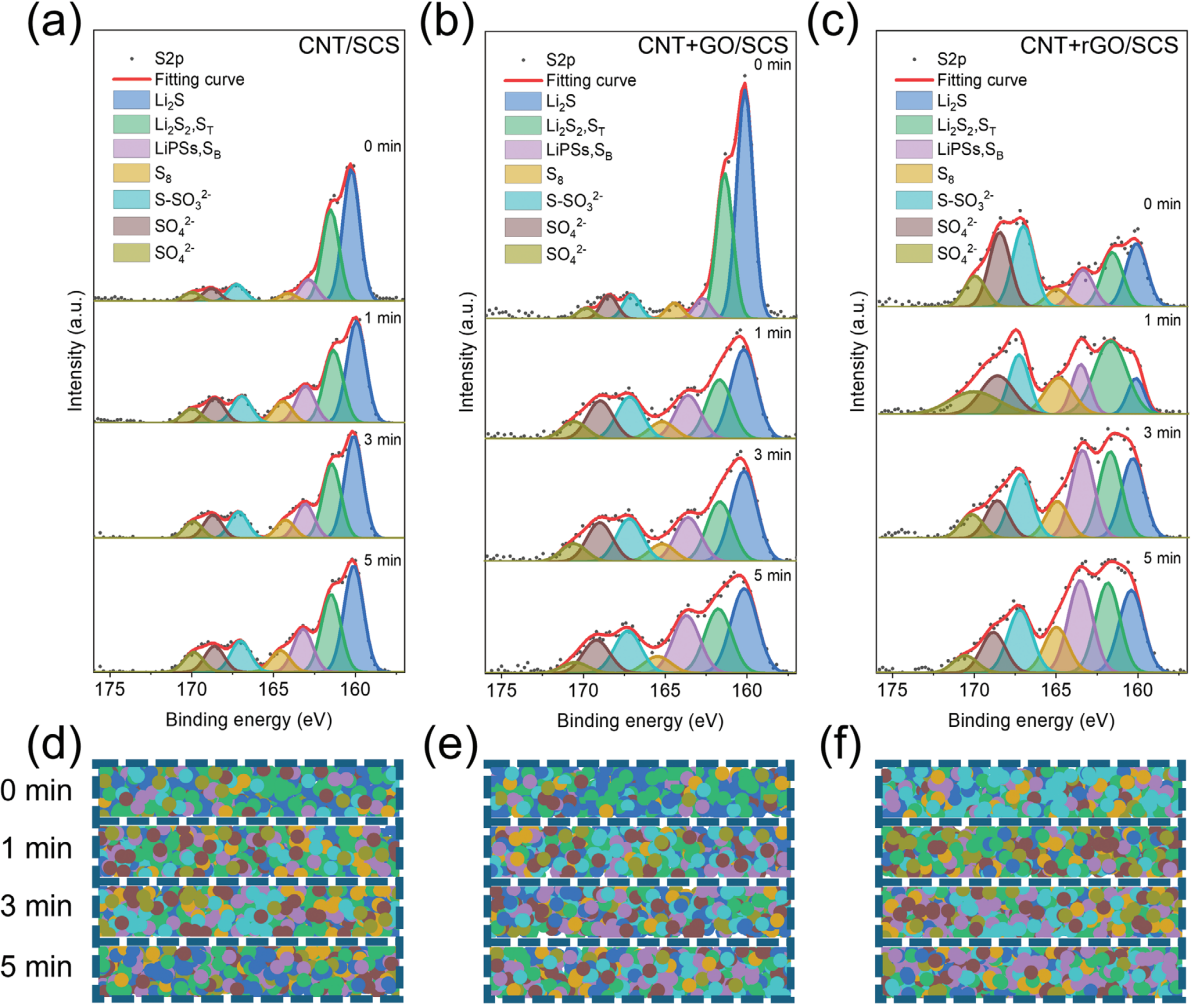
XPS S 2p spectra depth profile analysis of a) CNT/SCS, b) CNT+GO/SCS, and c) CNT+rGO/SCS configurations after cycling. The distribution maps of various sulfur species at different etching depths in d) CNT/SCS, e) CNT+GO/SCS, and f) CNT+rGO/SCS. The dot colors correspond to the species identified in the XPS spectra.

XPS S 2p spectra detailed deconvolution analysis was performed not only on the surface but at different depths (represented by etching time from 0 to 5 min) of the three current collectors after cycling. **Figure** **6**a–c shows significant differences in the intensity and distribution of the individual signals. First, strong signal peaks ≈160 eV represent the main active materials such as Li_2_S, Li_2_S_2_‐S_T_ (terminal sulfur), LiPSs‐S_B_ (bridging sulfur), and elemental sulfur,^[^ ^34^, ^36^ ^]^ indicating the ability of CNT‐supported current collectors to capture active sulfur species. Peaks ≈168 eV correspond to S‐SO_3_^2−^ and SO_4_^2−^ bonds,^[^ ^34^, ^36^ ^]^ originating from the decomposed products of the electrolyte, suggesting that these CNT membranes also adsorb by‐products from electrolyte decomposition. We plotted the area values of individual XPS peaks as concentration distribution maps at different etching depths using consistent colors for each species (Figure 6d–f). It is evident that in CNT+GO, the solid active material distribution (blue and green dots in Figure 6e) is mainly concentrated on the surface (0 min), indicating that polysulfides cannot effectively penetrate the deeper layers of CNT+GO fabric, resulting in high insulating lithium sulfide accumulation on the surface. In contrast, the signals in CNT+rGO show a more homogeneous distribution of active materials across all depth levels, with electrolyte decomposition primarily on the surface. More elemental sulfur (yellow dots) is also found at various depths due to more complete redox reactions. This demonstrates that CNT+rGO not only blocks decomposed electrolyte by‐products from entering the electrode during cycling but also effectively adsorbs polysulfides and converts them into solid lithium sulfide and sulfur active materials. In summary, the depth profiling and distribution maps indicate that using CNT+rGO as an advanced current collector provides the best active material redistribution effect for LSBs with the novel cell configuration consisting of sulfur‐coated separators and sulfur‐free current collectors.

## 3 Conclusion

In summary, our investigation into the novel cell configuration incorporating a sulfur‐coated separator and an advanced rGO/CNT interwoven fabric current collector for LSBs has yielded promising results. This configuration leverages the unique structural and electrochemical advantages of rGO/CNT composite substrate to significantly enhance the performance and stability of LSBs. The sulfur‐coated separator effectively utilizes the sulfur particles attached to its surface as the sole active material. This innovative approach ensures optimal sulfur utilization and mitigates the common issues of polysulfide dissolution and shuttle effect, which plague traditional sulfur cathodes. The superior surface morphology and adsorption capabilities of rGO/CNT current collectors highlight the addition of rGO, which enhances the adsorption sites and conductivity, resulting in more efficient polysulfide capture and conversion to lithium sulfide. This is further evidenced by the homogeneous distribution of active materials across various depths of the rGO/CNT fabric. Nitrogen adsorption‐desorption analysis confirmed that rGO/CNT boasts the highest surface area and optimal pore size distribution, which are critical for effective polysulfide capturing and lithium‐ion flow. This contributes to the stable and high‐capacity cycling performance observed. Electrochemical tests consistently demonstrated the superior kinetics and lower resistance of LSB with the CNT+rGO‐supported current collector. The symmetric cell tests and rate performance evaluations reaffirm its ability to maintain high capacity and stability across various cycling rates. A standout feature of the rGO/CNT current collector is its ability to maintain a homogeneous redistribution of active materials across different electrode depths. Post‐cycling XPS depth profiling reveals that the rGO/CNT configuration consistently retains active materials such as Li_2_S, Li_2_S_2_, and elemental sulfur throughout the electrode. Unlike other configurations where active materials accumulate on the surface, the rGO/CNT structure ensures deeper penetration and uniform distribution. This prevents the formation of an insulating lithium sulfide layer on the surface, contributing to more stable electrochemical reactions and improved overall battery efficiency. To further enhance this LSB configuration and reaction kinetics, future work could focus on: 1) improving contact between the active sulfur on the separator and the conductive current collector, 2) optimizing the porous structure and surface functionality of the current collector to enhance polysulfide absorption, and 3) developing a new electrolyte with higher polysulfide solubility. All in all, the novel LSB configuration with a sulfur‐coated separator and rGO/CNT current collector exhibits remarkable improvements in cycle life, capacity retention, and electrochemical performance. This advanced design not only enhances the efficiency of sulfur utilization but also addresses critical challenges in LSB technology, paving the way for more durable and high‐performance energy storage solutions.

## 4 Experimental Section

### Preparation of Sulfur‐Coated Separators

To prepare sulfur‐coated separators, sulfur powder (Showa Chemical) and polyvinylidene fluoride (PVDF; Arkema Kynar) binder were used in a weight ratio of 80:20. First, n‐methyl‐2‐pyrrolidone (NMP; Echo Chemical) and PVDF were stirred for 30 min. Then, sulfur was added and stirred for 4 h to ensure uniform mixing. The slurry was then uniformly coated onto the Celgard separator by a doctor's blade. The coated membrane was then placed in a vacuum oven set at 40 °C for 12 h of drying, which had an areal coating density of ≈2.0 – 3.2 mg cm^−2^.

### Preparation of CNT‐Supported Interwoven Fabrics

To prepare CNT‐supported current collectors, 0.1 g of CNT powder (Taiwan Carbon Materials Corp.) was dispersed in 50% isopropanol using 2 h of probe sonication for uniform dispersion. The suspension was vacuum‐filtered, and the resulting CNT fabrics were dried at 40 °C under vacuum for 72 h. Once dried, the free‐standing CNT substrates were cut into 15 mm diameter circular current collectors. For GO surface modification, GO (The Sixth Element) was added to the CNT dispersion at a 10:90 weight ratio, followed by sonication and vacuum filtration to form free‐standing interwoven fabrics. For rGO surface modification, the CNT+GO mixture was dried at 40 °C for one week, thermally reduced at 950 °C under argon in a tube furnace, and then redispersed in suspension by sonication followed by vacuum filtration to form self‐supporting substrates.

### Material Characterizations

The microstructure of the samples was observed using a SEM tool (Hitachi SU‐8010). Prior to imaging, the samples were coated with platinum using a sputter coater to enhance surface conductivity and avoid charging. Raman spectroscopy (Princeton SP2750), with a laser wavelength of 532 nm and a scanning range of 800–2000 cm^−1^, was employed to analyze carbon‐based materials. Nitrogen adsorption–desorption isotherms at 77 K were measured using a gas adsorption analyzer (Anton Paar Autosorb iQ) to elucidate porous structure. The surface area was calculated using the BET method, while the pore size distribution and pore volume were determined using the DFT method. The UV–vis; Jasco V‐370 spectroscopy was used to detect the adsorption behavior of soluble polysulfides. XPS (ULVAC‐PHI Quantera II) depth profiling was utilized to analyze the composition and redistribution of materials after cycling. The XPS measurements were conducted with an Al Kα monochromatic X‐ray source (1486.8 eV).

### Cell Assembly and Electrochemical Analysis

CR2032 coin cells were used for testing. The prepared CNT‐supported current collector was placed at the center of the battery case, and electrolyte was added to wet the substrate. Next, the sulfur‐coated separator membrane was aligned with the CNT fabrics and laid flat to adhere to the CNT surface. Additional electrolyte was then added, followed by placing a lithium metal foil as the counter electrode. All assembly processes were conducted inside a glove box filled with argon, maintaining moisture and oxygen levels at ≤ 0.5 ppm. Battery testing was conducted using a programmable battery testing system (Neware). Before each test, batteries rested for 12 h on the instrument. The first three cycles consisted of discharging to 1.8 V and charging to 2.8 V at 0.1C. Subsequent cycles were assessed at 0.3C within the same voltage window. The theoretical specific capacity was calculated as 1675 mAh g^−1^ for 1C. Cyclic voltammetry (CV; BioLogic SP‐50e) was performed by scanning from open‐circuit voltage (OCV), with a rate fixed at 0.1 mV s^−1^ within a potential range of 1.5–2.8 V. Electrochemical impedance spectroscopy (EIS) utilized the same potentiostat, applying a voltage magnitude of 10 mV over a frequency range of 1 MHz–10 mHz for analysis.

## Conflict of Interest

The authors declare no conflict of interest.

## Supporting information



Supporting Information

## Data Availability

The data that support the findings of this study are available from the corresponding author upon reasonable request.
