# Supplementary material for: Advanced Li–S Battery Configuration Featuring Sulfur‐Coated Separator and Interwoven rGO/CNT Fabric Current Collector
Source: Small. 2024 Oct 29;21(8):2405365. doi: 10.1002/smll.202405365 (PMC11855221; doi:10.1002/smll.202405365)
Supplement: Supplementary file 1 — Supporting Information [file SMLL-21-2405365-s001.docx]

**Supporting Information**

**Advanced Li-S Battery Configuration Featuring Sulfur-Coated Separator and Interwoven rGO/CNT Fabric Current Collector**

Kuan-Cheng Chiu^1^, Asif Latief Bhat^1^, Ching-Kuan Yang^2^, Sheng-Heng Chung^3^, Niall Tumilty^1^ and Yu-Sheng Su*^1,2^

^1^International College of Semiconductor Technology, National Yang Ming Chiao Tung University, 1001 Daxue Road, Hsinchu City 300093, Taiwan

^2^Industry Academia Innovation School, National Yang Ming Chiao Tung University, 1001 Daxue Road, Hsinchu City 300093, Taiwan

^3^Department of Materials Science and Engineering, National Cheng Kung University, No.1 University Road, Tainan City 70101, Taiwan


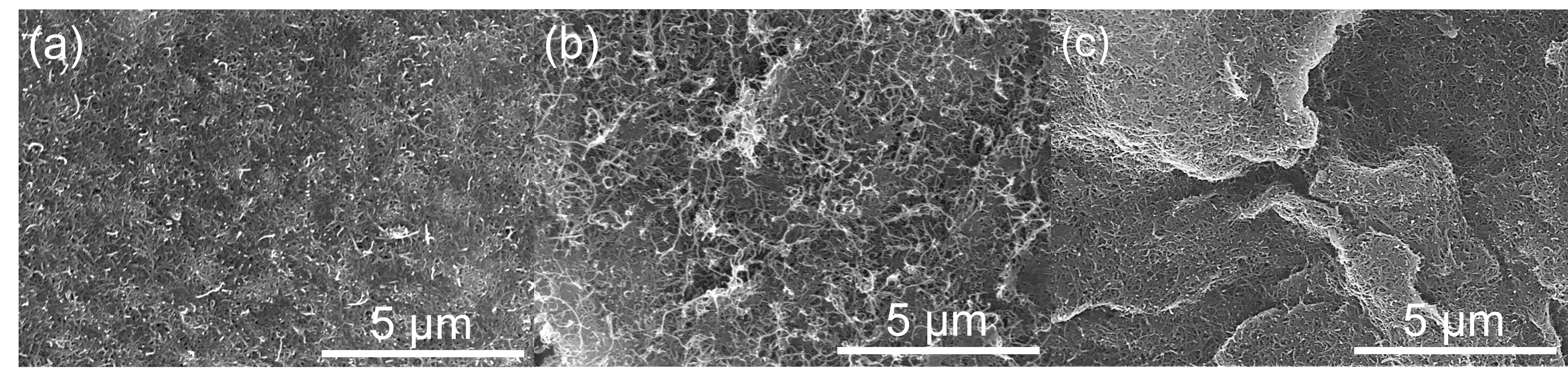


**Figure S1.** SEM images of different CNT-supported current collectors before cycling: (a) CNT, (b) CNT+GO, and (c) CNT+rGO.


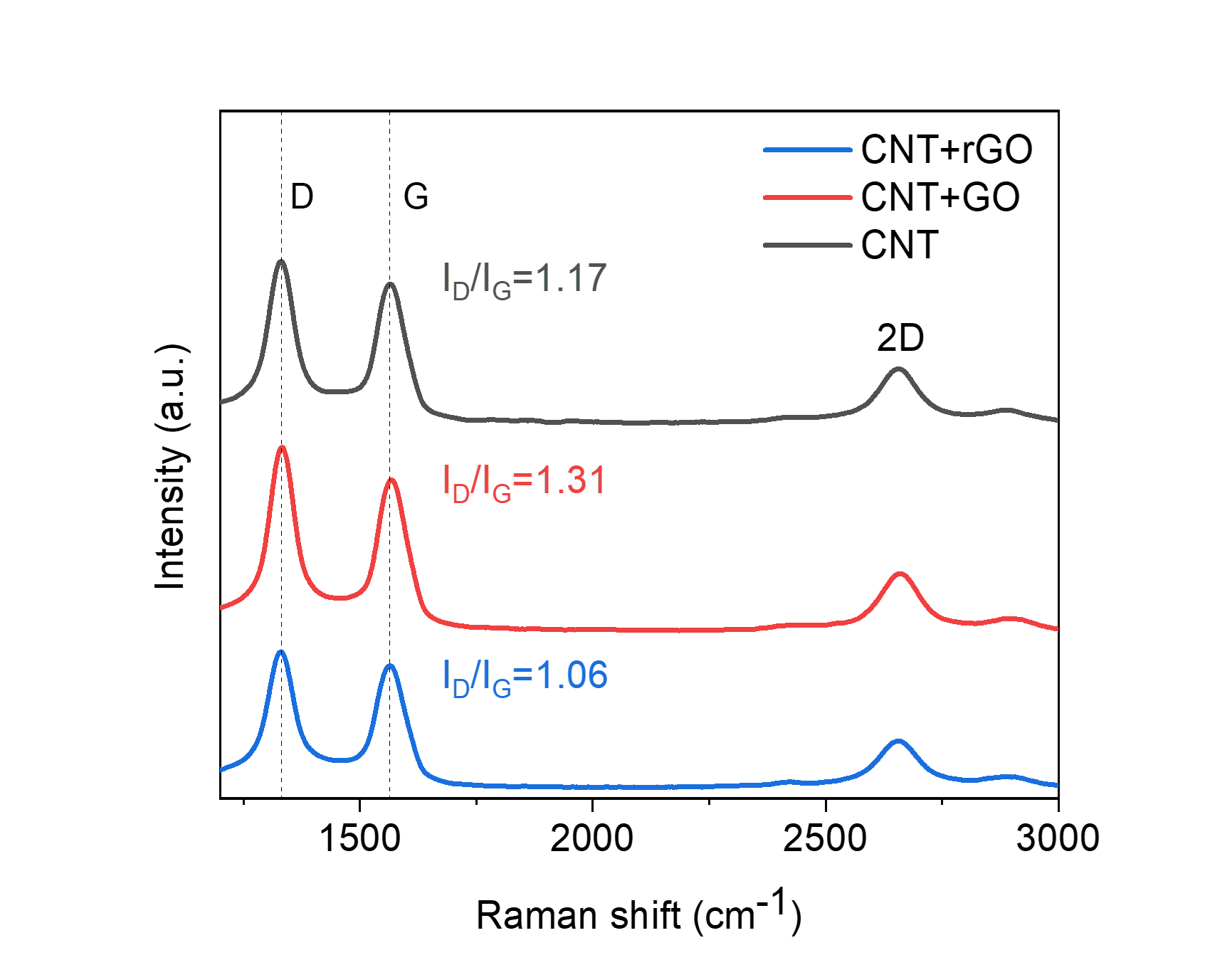


**Figure S2.** Raman spectra of different CNT-supported current collectors.


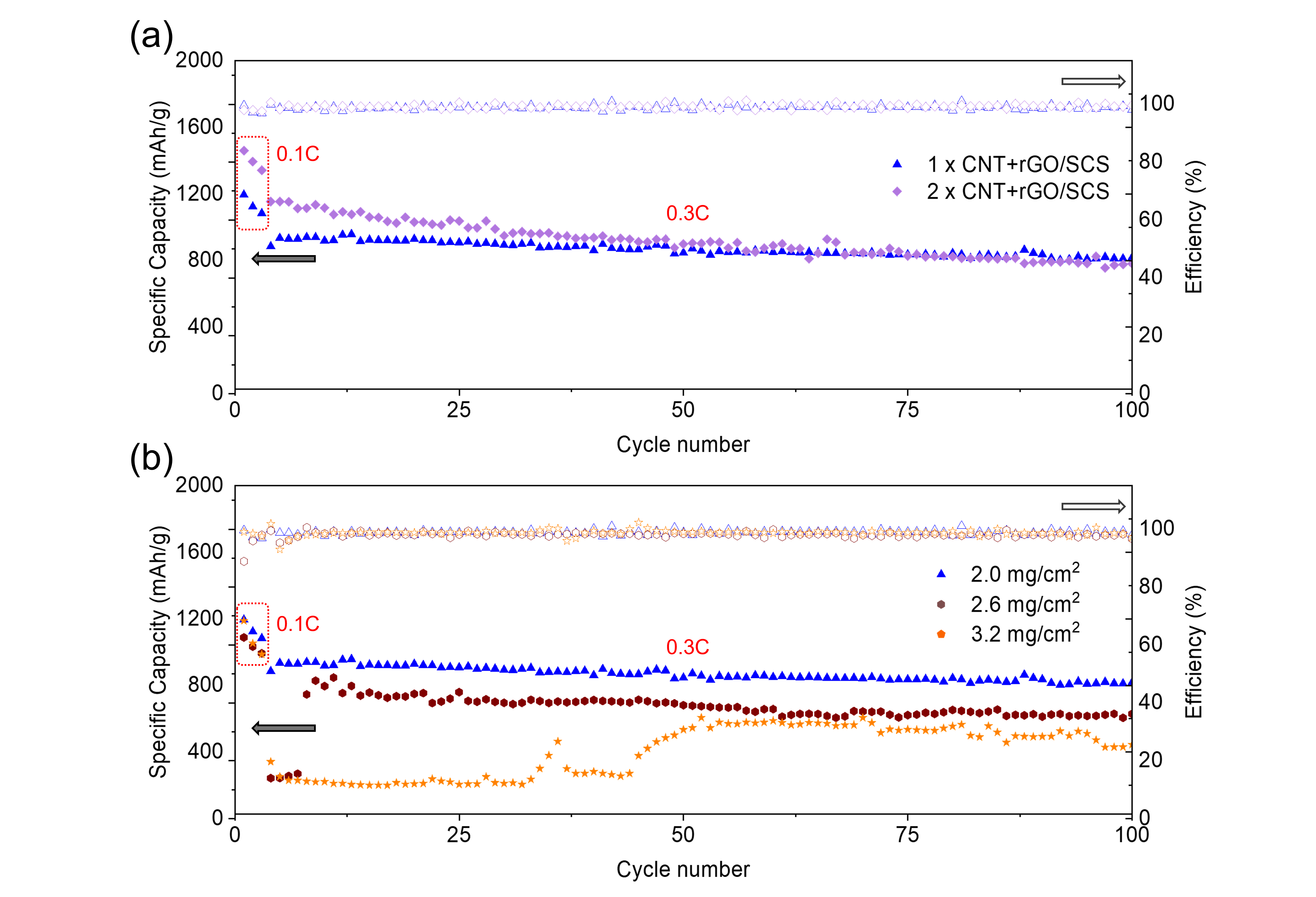


**Figure S3.** (a) Cycle life with the sulfur-coated separator and different number of layers of CNT+rGO current collector. (b) Cycle life with the sulfur-coated separator with different active material laodings and a CNT+rGO current collector.


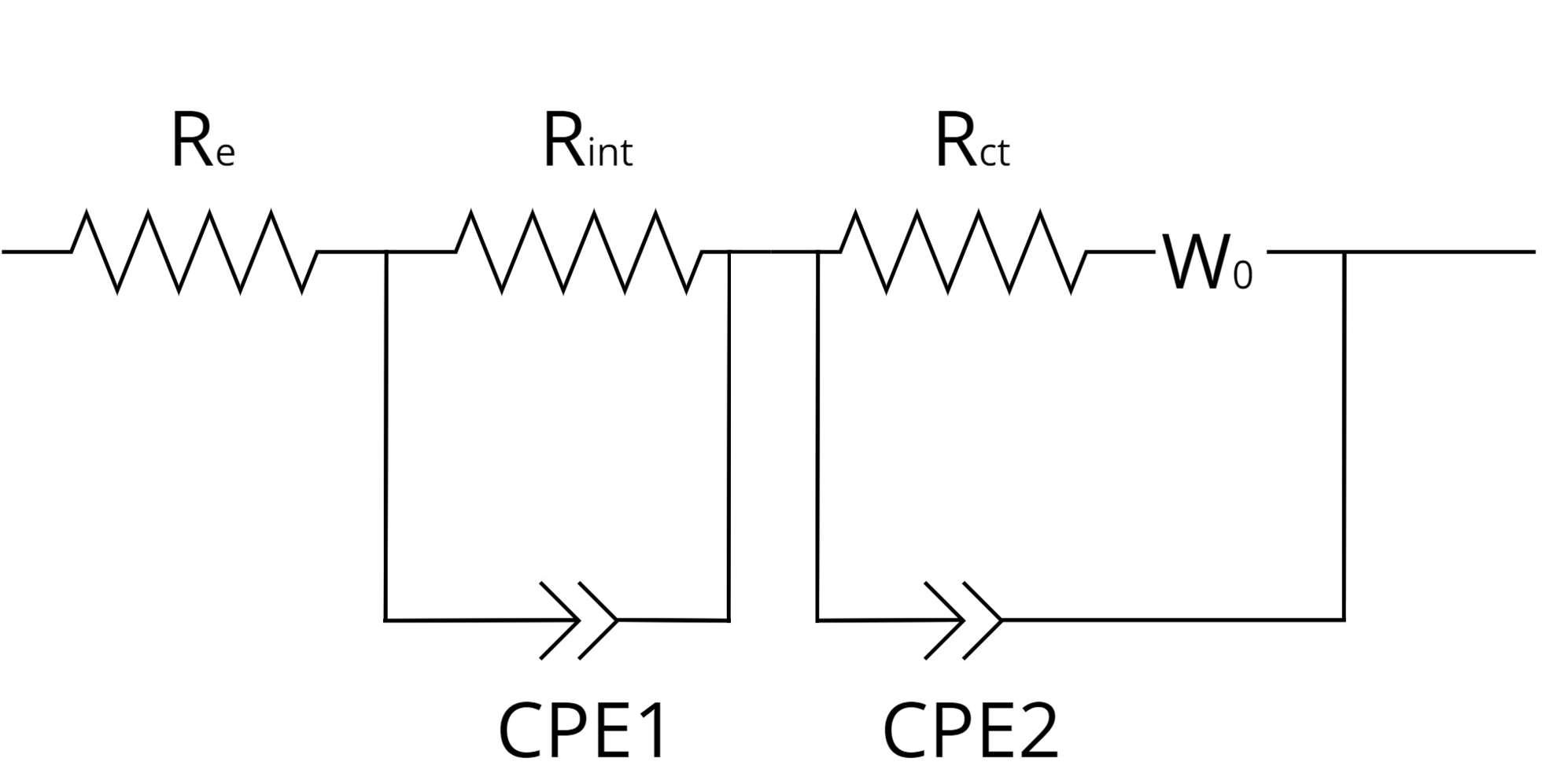


**Figure S4.** Equivalent circuit diagram for EIS analysis.


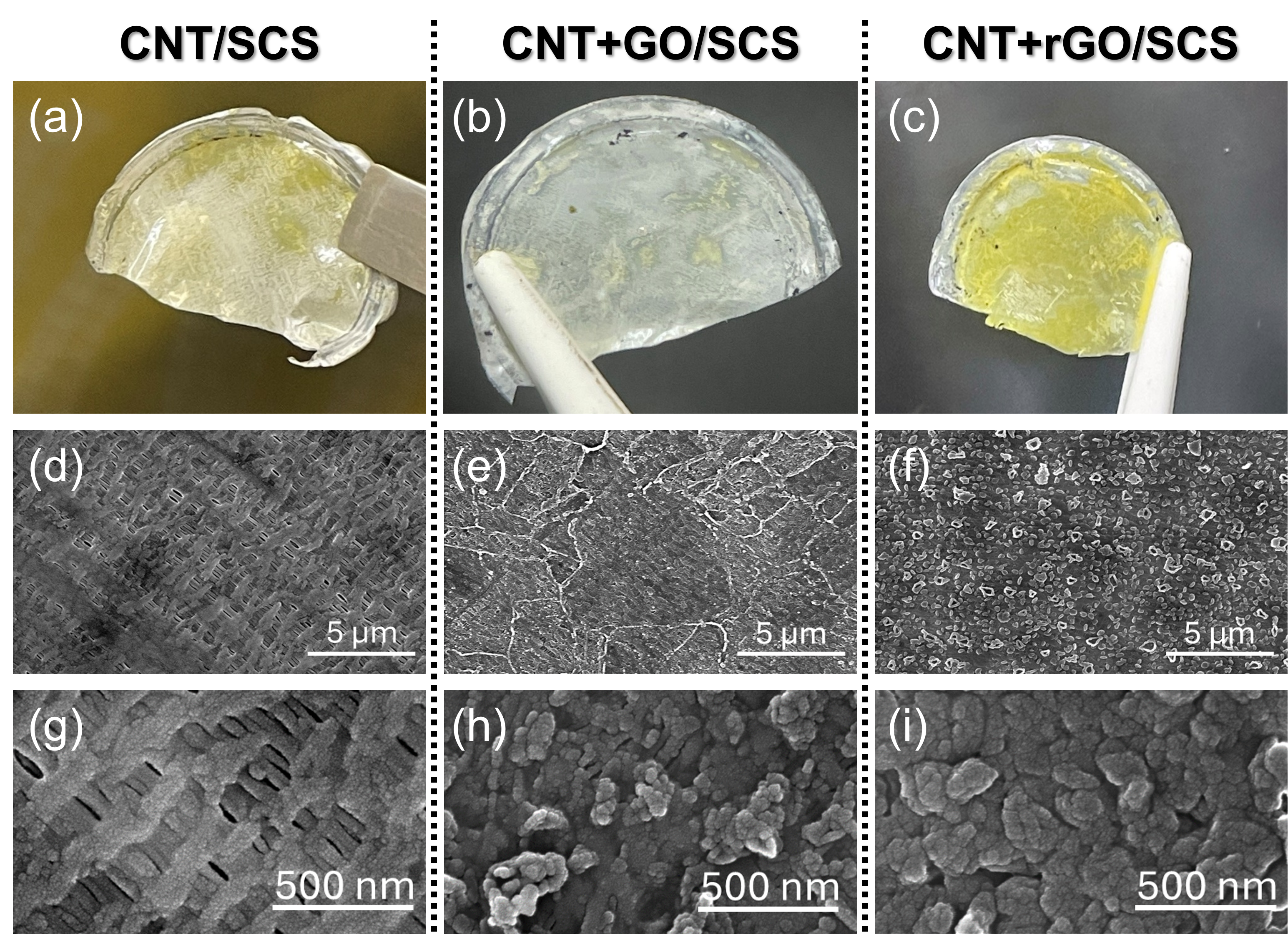


**Figure S5.** (a-c) Optical images of the separators with different CNT-supported current collectors after cycling. (d-i) SEM images of the separators with different CNT-supported current collectors after cycling.


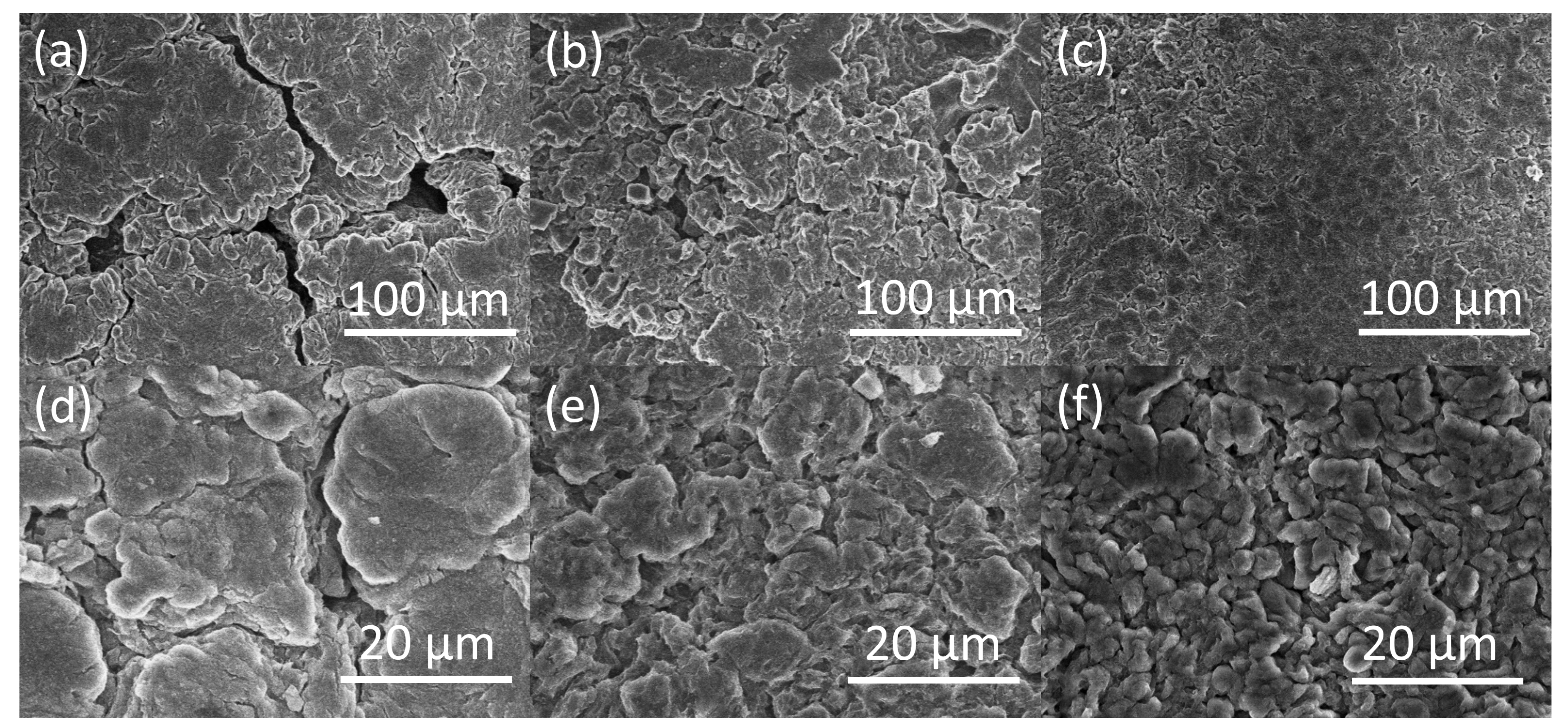


**Figure S6.** SEM images of the Li electrodes with (a,d) CNT/SCS, (b,e) CNT+GO/SCS, and (c,f) CNT+rGO/SCS configurations after cycling.


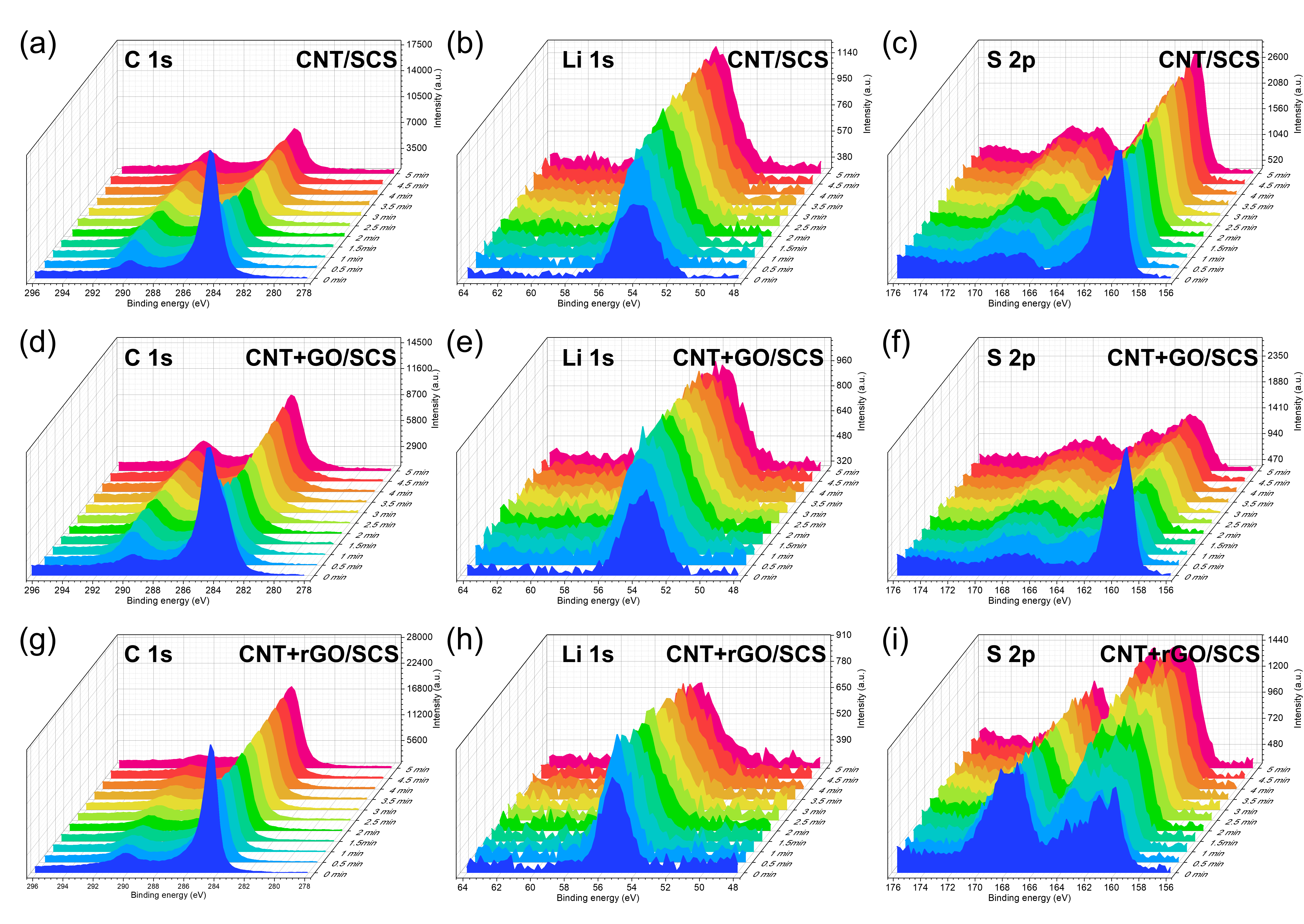


**Figure S7.** XPS depth profile analysis of different CNT-supported current collectors with sulfur-coated separators after cycling. (a) C 1s, (b) Li 1s, (c) S 2p spectra in CNT/SCS; (d) C 1s, (e) Li 1s, (f) S 2p spectra in CNT+GO/SCS; and (g) C 1s, (h) Li 1s, (i) S 2p spectra in CNT+rGO/SCS.
